# Cladosporols and PPARγ: Same Gun, Same Bullet, More Targets

**DOI:** 10.3390/biom14080998

**Published:** 2024-08-13

**Authors:** Roberta Rapuano, Antonella Mercuri, Sabrina Dallavalle, Salvatore Moricca, Antonio Lavecchia, Angelo Lupo

**Affiliations:** 1 Dipartimento di Scienze e Tecnologie, Università del Sannio, Via dei Mulini, 82100 Benevento, Italy; rrapuano@unisannio.it (R.R.); a.mercuri1@studenti.unisannio.it (A.M.); 2 Dipartimento di Scienze per gli Alimenti, la Nutrizione e l’Ambiente, Università degli Studi di Milano, Via Celoria 2, 20133 Milano, Italy; sabrina.dallavalle@unimi.it; 3 Dipartimento di Scienze e Tecnologie Agrarie, Alimentari, Ambientali e Forestali (DAGRI), Università degli Studi di Firenze, Piazzale delle Cascine 28, 50144 Firenze, Italy; salvatore.moricca@unifi.it; 4 Dipartimento di Farmacia “Drug Discovery Laboratory”, Università di Napoli “Federico II”, Via D. Montesano 49, 80131 Napoli, Italy

**Keywords:** Cladosporols, PPARγ agonists, proliferation, migration, apoptosis, lipogenesis

## Abstract

Several natural compounds have been found to act as PPARγ agonists, thus regulating numerous biological processes, including the metabolism of carbohydrates and lipids, cell proliferation and differentiation, angiogenesis, and inflammation. Recently, Cladosporols, secondary metabolites purified from the fungus *Cladosporium tenuissimum*, have been demonstrated to display an efficient ability to control cell proliferation in human colorectal and prostate cancer cells through a PPARγ-mediated modulation of gene expression. In addition, Cladosporols exhibited a strong anti-adipogenetic activity in 3T3-L1 murine preadipocytes, preventing their in vitro differentiation into mature adipocytes. These data interestingly point out that the interaction between Cladosporols and PPARγ, in the milieu of different cells or tissues, might generate a wide range of beneficial effects for the entire organism affected by diabetes, obesity, inflammation, and cancer. This review explores the molecular mechanisms by which the Cladosporol/PPARγ complex may simultaneously interfere with a dysregulated lipid metabolism and cancer promotion and progression, highlighting the potential therapeutic benefits of Cladosporols for human health.

## 1. PPARγ: Gene Structure, mRNAs, Proteins, and Related Functions

**Figure 1:**
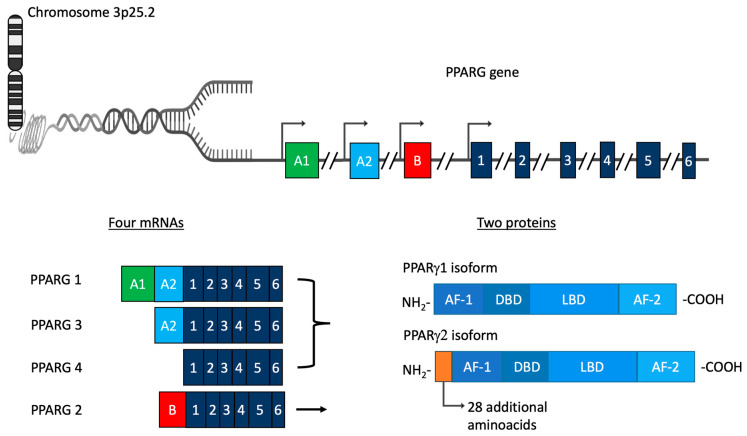
Schematic representation of PPARγ gene, mRNA species, and protein isoforms.

Peroxisome Proliferator-Activated Receptors (PPARs) belong to nuclear receptor class 2 and usually remain inactive in the nucleus until they get in touch with the ligand to which they bind. Three members of its own family, i.e., PPARα (NR1C1), PPARβ/δ NR1C2), and PPARγ (NR1C3), are included [1,2]. Similar to other nuclear receptors, the protein sequence of PPARs includes three different structural and functional domains: the N-terminal transactivation domain (AF1), the DNA-binding domain (DBD) and the C-terminal ligand binding domain, which contains the ligand-dependent transactivation function (AF2). The interaction of the ligand with the LBD (ligand binding domain) of the PPARs generates, in its structure, a conformational change that, in turn, is responsible for heterodimerization with the Retinoid X receptor (RXR) and, sequentially, the migration of the activated receptor in the nucleus and the binding to the PPAR response element (PPRE) on the DNA. This binding stimulates the transcription of various gene targets and, consequently, the regulation of various biological processes [3]. Although PPARs show structural analogies, they display distinctive functions due to different tissue distributions, a differential response to specific ligands, and, finally, diverse biochemical features. PPARα, which is present in the liver, heart, and skeletal muscle, stimulates the fatty acid transport and oxidation and, in such a way, regulates lipid metabolism [4]. PPARβ/δ, which is expressed in the skeletal muscle, adipocytes, macrophages, skin, and gastrointestinal tissue, regulates lipid metabolism, but it is also involved in keratinocyte differentiation and macrophage-derived inflammation [5]. Finally, PPARγ, which is mainly expressed in the adipose tissue, immune cells, and colon, is the master gene of adipocyte differentiation and plays a significant role in controlling insulin resistance and energy homeostasis [6]. The complexity of the PPAR family is, then, further increased as a result of the demonstration that the four distinctive mRNAs originated in humans from the same PPARγ gene through the use of different promoters and alternative splicing. Among these, PPARγ1, PPARγ3, and PPARγ4 mRNAs produce the same protein product, namely, PPARγ1 (477 aa), while PPARγ2 mRNA generates the PPARγ2 isoform containing 28 additional amino acids at the N-terminus (505 aa) (Figure 1) [6]. While the PPARγ1 isoform is expressed in various tissues including the skeletal muscle; pancreas; heart; colon; white (WAT) and brown adipose tissue (BAT); immune cells, such as macrophages; and kidney, PPARγ2 is strongly restricted to the adipose tissue, although a high-fat diet may also induce it in other tissues [7,8]. Due to the characteristic tissue distribution, PPARγ is considered to be the master regulatory gene of adipocyte differentiation, playing a fundamental role in fatty acid synthesis and the transport and formation of triglyceride depots [9]. Indeed, the lack of adipose tissue in a PPARγ-null mouse definitively confirmed the crucial function of PPARγ in this tissue [10]. Additionally, PPARγ expression was also verified within the WAT perivascular niche in an adipocyte ancestor population, and this discovery indicated that it may display a crucial function in adipocyte self renewal [11]. Finally, maintaining a mature adipocyte phenotype has been shown to require the presence of PPARγ, since its selective removal in this cell type led to survival for only a few days [12]. Beyond its function in lipid metabolism and adipocyte differentiation, PPARγ regulates the expression of genes controlling glucose homeostasis and uptake, including Glut1, Glut4, and the c-Cbl–associated protein (CAP) [13,14]. Moreover, PPARγ modulates the synthesis and secretion of various adipokines, such as adiponectin, resistin, leptin, and tumor necrosis factor-α (TNF-α, which controls insulin sensitivity [15,16,17,18]. Recent studies have shown that PPARγ can induce the expression of adipose-related factors FGF1 and FGF21, members of the fibroblast growth factor family, which are able to maintain adipose homeostasis and counteract insulin resistance [19,20]. Intriguingly, these factors can act in both autocrine and paracrine manners, locally transmitting PPARγ signals to stimulate adipogenesis and sustain the insulin response [20]. It is very interesting that the distinct PPAR isoforms, despite their structural and functional similarities, can display peculiar properties due to their specific tissue distribution and selective responses to different ligands, thus generating such a wide range of actions. In accordance with this reasoning, PPARγ is, therefore, able to activate the expression of different genes controlling glucose homeostasis including Glut1, Glut4, and CAP, but at the same time PPARγ also modulates the synthesis and secretion of different adipokines (adiponectin, leptin, and TNF-α) from the adipose tissue, thus enhancing the insulin response. Therefore, it is reasonable to argue that, through PPARγ activation, different protein factors might stimulate distinctive pathways that, in turn, lead to improved glucose homeostasis. A PPARγ-driven targeted therapeutical approach requires selective ligands that activate the receptor, resulting in beneficial effects in fighting diabetes and obesity.

Furthermore, PPARγ is involved in the differentiation of immune cells (antigen-presenting myeloid dendritic cells and macrophages) and in the stimulation of both the immunological response and metabolic processes. Indeed, in dendritic cells, the activated PPARγ modulates lipid transport and metabolism, but also stimulates antigen uptake, dendritic differentiation, cytokine production, and antigen presentation [21]. In macrophages, PPARγ is involved in lipid metabolism and the differentiation of monocytes into macrophages [22]. Additionally, PPARγ is also involved in the polarization of M1 to M2 macrophages, thus blocking the secretion of proinflammatory cytokines, such as TNF-α, interleukin-1β (IL-1β), and interleukin-6 (IL-6), and promoting anti-inflammatory effects [23]. This is achieved through a transrepression mechanism, which activates inflammatory genes such as NF-kB and AP-1 in the M1 macrophage.

Several studies demonstrated that PPARγ can inhibit angiogenesis during the tumor formation. For example, thiazolidinediones-activated PPARγ is able to arrest tumor angiogenesis and in vivo cell growth in ovarian carcinoma and pancreatic cancer [24,25]. In gliomas, a sustained PPARγ expression has been shown to positively correlate with thrombospondin-1 (TSP-1) expression [26]. TSP-1, a glycoprotein present in the extracellular matrix and involved in the growth and adhesion of cancer and epithelial cells, counteracts angiogenesis also inhibiting tumor growth and metastasis formation [26].

It is well known that the dysfunction of nuclear receptors (NRs), due to their over-expression or under-expression, mutations, truncations, post-transductional modifications, and other events modifying the structure and the function of NRs, can induce the activation of different pathways and provoke the initiation and promotion of several cancers. Understanding of these complicated processes usually provides unexpected solutions in the therapeutic treatment of tumor patients. The expression of PPARγ has been reported in various tumor tissues and cell lines. Targeting PPARγ, indeed, through its activation mediated by specific agonists, displayed an efficient cell growth inhibition [27,28]. As mentioned above, PPARγ is particularly expressed in the colon, where it plays a protective role in in vitro and in vivo colorectal cancer (CRC) models [29,30,31]. In the gastrointestinal tract, in fact, PPARγ inhibits cellular proliferation, promotes differentiation, and stimulates apoptosis [32]. Several studies in mice and humans show that PPARγ prevents tumor initiation and progression, acting as a tumor suppressor gene [33]. In CRC-derived cells and transplanted tumors in nude mice, PPARγ activation, after the administration of selective ligands such as thiazolinediones (TZDs), provoked cell growth inhibition, G0/G1 arrest, and caspase-activated apoptosis [34,35].

It is reasonable to think that the multiple PPARγ functions depend on the distinctive binding mode of the selective ligands to the receptor ligand binding domain (LBD), which causes the recruitment of distinct protein cofactors capable, in turn, of activating different pathways involved in the control of metabolism, proliferation, or differentiation. The choice to activate (or not) these pathways is also likely to be influenced by the amount of the available receptors in a given cell, the chemical features of the ligands, and their affinity for the receptor. Ligands can induce conformational changes in the PPARγ LBD, thus affecting the recruitment of functional effectors (coactivators, corepressors, molecular adapters, chromatin modifying enzyme activities, etc.), which, in turn, facilitates chromatin opening and the activation of numerous target genes.

## 2. PPARγ Activation by Natural and Synthetic Ligands

Although the first pioneering studies about PPARs date back to the 1950s, it was necessary to wait until the 1990s to finally confirm the existence of PPARs through cloning experiments carried out in the laboratory of Issemann and Green [36]. Afterwards, the hunt started for finding natural molecules that bind specifically to PPARs, considered as biological sensors capable of detecting environmental changes and developing an adaptive response via modulated gene expression. Among the endogenous agonists of PPARs, the first natural molecules were fatty acids and their derivatives, which are usually produced by de novo lipogenesis, derived from lipolysis, or provided by the diet [37]. Dietary lipids, including unsaturated fatty acids produced from plants, are able to activate all three types of PPARs. Phospholipids usually activate PPARα, whereas 15d-PGJ2 prefers PPARγ and prostacyclin I2 binds to PPARβ/δ. Many studies have been carried out to identify other endogenous lipids, such as PPARγ ligands, but it has been difficult to define the mechanisms by which these molecules can activate the receptor and generate biological effects on lipid metabolism [38,39]. On the contrary, searching synthetic ligands for PPARγ was a more productive work, leading to the discovery of thiazolinediones (TZDs), potent activators of PPARγ with an elevated ability to sensitize cells to insulin stimulation [40]. In 1983, Ciglitazone was discovered as the first molecule displaying a certain ability of insulin sensitizing, but its clinical use has never been approved because of its weak therapeutic effect on diabetics [41]. Among TZDs, Rosiglitazone (BRL49653) was the first shown to induce the differentiation of C3H10T1/2 stem cells into adipocytes. This was, indeed, the first experimental evidence that PPARγ is a TZD target [42]. Later, Troglitazone was synthesized and used in therapeutical protocols to sensitize diabetic patients to insulin, stimulating glucose transport and metabolism. Afterwards, Pioglitazone followed and, together with Rosiglitazone, was definitively approved for the clinical treatment of Type 2 Diabetes Mellitus (T2DM) patients in 1999. Unfortunately, the oral administration of these molecules, which are able to strongly activate PPARγ, provoked unwanted side effects at the same time (weight gain, fluid retention, osteoporosis, cardiovascular diseases, and bladder cancer) [43,44,45,46]. For these reasons, both Rosiglitazone and Pioglitazone were removed from the market in the United States and Europe [47,48]. Despite these drawbacks, TZDs have significantly advanced our understanding of diabetes management, particularly T2DM. New powerful and sophisticated technologies promise to clarify every single aspect of PPARγ activation and signaling in tissues to regulate different and vital processes. Many efforts will be led to develop novel molecules that will reduce or eliminate the adverse effects associated with TZDs and improve insulin sensitization through new PPARγ-mediated molecular mechanisms.

**Figure 2:**
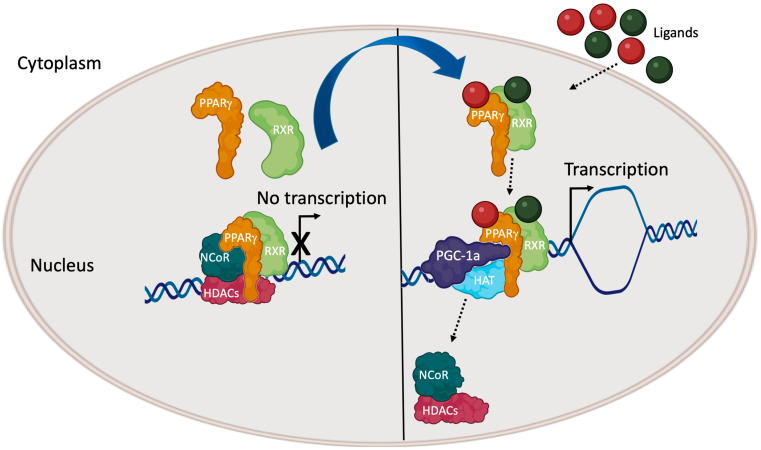
Molecular mechanisms of the transactivation of PPARγ-mediated gene transcription.

PPARγ requires ligand binding to express its properties, inducing conformational changes that facilitate the activated receptor entry into the nucleus. Hence, PPARγ heterodimerizes with RXR, which, in turn, is also bound by its specific ligand and, together as the PPARγ/RXR complex, they recognize the PPRE motif present in the promoter regions of several gene targets, activating their transcription. In the absence of a ligand, the PPARγ/RXR complex associates with co-repressors like NCoR (nuclear receptor corepressor 1) and SMRT (silencing mediator of retinoic acid and thyroid hormone receptor), inhibiting PPARγ-mediated transcription. When an agonist binds to PPARγ, co-repressors are removed from the PPARγ/RXR complex and co-activators are recruited. Co-activators, such as CBP (CREB-binding protein), MED1 (Mediator 1), SRC1 (steroid receptor coactivator1), SRC2, SRC3, and PGC1α (peroxisome proliferator-activated receptor gamma coactivator 1α), closely interact with the ternary PPRE/PPARγ/RXR complex-promoting transcription through modifying the chromatin structure and recruiting transcription factors to the promoters of gene targets (Figure 2) [49]. Understanding how PPARγ ligands enhance the signal transduction of the activated receptor and sustain the target gene transcription requires focusing the binding mode of these molecules to the PPARγ LBD.

## 3. PPARγ Full Agonists, Partial Agonists, and Antagonists: Distinctive Modes of Binding

**Figure 3:**
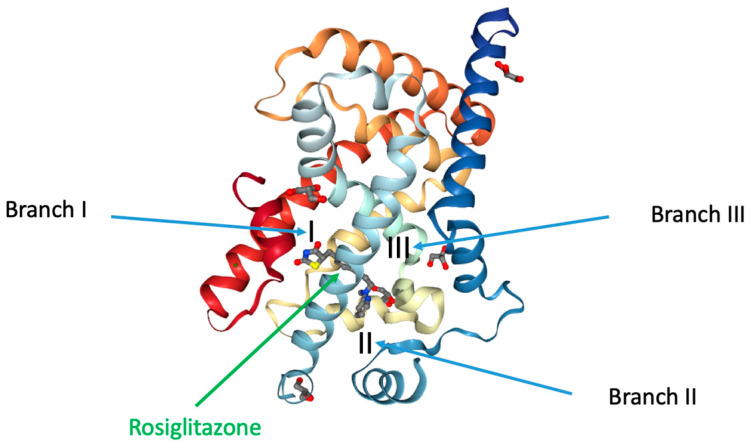
Localization of the different branches in the PPARγ LBD. The branches of the ligand binding pocket have been labelled with Roman numerals. The interaction of full agonist Rosiglitazone to PPARγ is also reported. Rosiglitazone is located around helix 3 (PDB-4XLD modified by Lupo et al.).

Similar to most nuclear receptors, beyond the other structural and functional domains, PPARγ shows a highly complex and conserved LBD at the C-terminus. Within this large domain (formed by about 270 amino acids) are included four subdomains displaying different functions. Firstly, there is an additional dimerization module, which is different from the one in the DNA-binding domain and works in a DNA-dependent manner. Afterwards, within the LBD is located the ligand binding pocket of PPARγ, which stabilizes the structure of the domain [50]. Thirdly, a surface necessary to recognize and bind to transcriptional co-regulators has been revealed. Finally, a fourth subdomain was found in the LBD, bearing the activation function 2 (AF2), which is responsible of the stabilization of the ligand-activated PPARγ, permitting the interaction with transcriptional co-regulators. Among these functions, the one showing the most evident differences, also compared to other nuclear receptors, is properly the ligand binding pocket. Both the primary structure and volume of the cavity of the PPARγ LBD pocket are really different from the classic nuclear receptors and even other PPAR family members, suggesting that in the tridimensional structure of the PPARγ ligand binding pocket resides a specific sensitivity towards certain ligands. Indeed, the PPARγ ligand binding pocket is the largest among the other nuclear receptors. The fundamental work of Nolte’s group in 1998 confirmed these previous details and proposed additional new evidences about the PPARγ LBD pocket, which proved to be truly important to understand its function [51]. On the basis of this study, the first X-ray crystal structure of PPARγ was obtained, and, hence, we learned about many features of the PPARγ ligand binding pocket. For example, now we know that this subdomain occupies the center of the PPARγ LBD and can be described as a large Y- or T-shaped cavity (its size is approximately 1200 Å^3^) formed by 13 α-helices and four β-sheet strands. It is divided into three branches with different abilities and specific sensitivities towards various ligands (Figure 3). The first branch, surrounded by the H3, H5, H11, and H12 helices, displays a hydrophilic feature that interacts with the acidic head group of ligands like Rosiglitazone, a well-known member of the thiazolidinedione TZD family. Branch I is located very close to the H12 helix, which is part of the activation function-2 (AF-2) co-regulator binding surface and is stabilized through ligand-dependent conformational changes. Branch II, formed by the H2′, H3, H6, and H7 helices and a β-sheet, is, instead, strongly hydrophobic. Branch III, finally, delimited by a β-sheet and the H2, H3, and H5 helices, has both hydrophilic and hydrophobic features. Understanding the differential structures within the PPARγ ligand binding pocket has been crucial in identifying how various ligands interact with it and elicit specific biological responses. Researchers have extensively studied which ligands enter each branch and how they bind to specific side chains of amino acids within the pocket. These interactions ultimately determine the ligand’s biological effects and have guided the development of new PPARγ ligands with peculiar desired actions.

**Figure 4:**
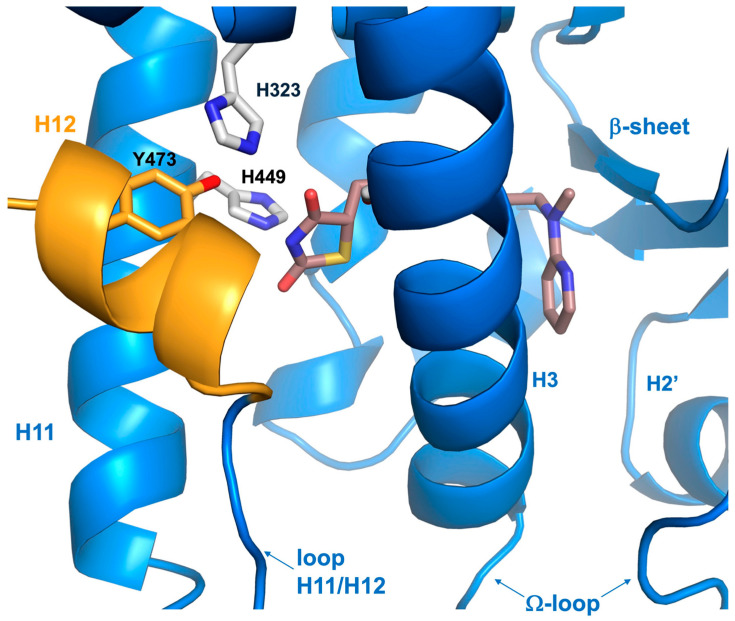
Crystal structure of Rosiglitazone bound to the PPARγ LBD (PDB: 2PRG). H12 is shown in orange. The Ω–loop, a flexible loop region between H2′ and H3, and the β-sheet region of the LBD are displayed.

Starting from the early 2000s, the clinical use of TZDs as activators of PPARγ has significantly impacted the management of dyslipidemia and diabetes [52]. Rosiglitazone, the first TZD used in patients affected by T2DM, was demonstrated to show high efficiency in enhancing insulin sensitivity and controlling the peripheral uptake of glucose [53]. In addition, Rosiglitazone treatment caused a strong reduction in circulating fatty acids and other proteins involved in cardiovascular disease [54]. Other members of the TZD family have been, successively, used as insulin sensitizers in the treatment of T2DM patients, raising great hopes for a targeted diabetes therapy. The thiazolidinedione head group is a crucial feature of TZDs for entry and binding to the hydrophilic part of branch I, limited by the H3, H5, H6, and H12 helices. Rosiglitazone, fitting this model perfectly, enters the PPARγ cavity and binds to the side chains of certain amino acids within the LBD pocket, stabilizing the fluctuating and dynamic H12 helix through hydrogen bonds residues H323, H449, and Y473 on the inner surface of helix 12 (Figure 4). This stabilization promotes the engagement of coactivators and PPARγ transactivation [55]. Rosiglitazone contacts other areas of the PPARγ binding pocket, turning all around the H3 helix and placing itself perpendicular to it. In such a way, Rosiglitazone also increases binding affinity through hydrophobic interactions and van der Walls contacts with amino acid residues of H3, H5, H6, and H7 and the β-sheet lying on branches II and III of the PPARγ binding pocket [55]. Ligands like Rosiglitazone, which bind to the same subdomain of the PPARγ pocket with the same mechanism, have been defined as PPARγ full agonists. In transient transfection assays carried out in HEK293 cells (which do not express any type of PPARγ), only in the presence of ectopic receptors and chimeric plasmids bearing PPRE motifs upstream of the reporter luciferase gene, PPARγ full agonists display maximal PPARγ–induced transcriptional activity. This suggests that they are able to elicit and organize a stable surface onto the AF2 domain for the coactivator recruitment in the proximity of most PPARγ target genes. Additional TZD members, such as Troglidazone, Pioglitazone, Rivoglitazone, and variants of the same pharmacocore were used in the treatment of T2DM patients, and all behave as PPARγ full agonists. Although they showed a strong potential as insulin sensitizers in T2DM patients, due to their high affinity for binding to PPARγ, they were withdrawn from market, because different adverse side effects in TZD-treated patients affected by T2DM have been evidenced. Among these, renal fluid retention, heart failure, bone fractures, hepatotoxicity, and cancer raised particular interest and, at the same time, strong concern. Indeed, observational trials focusing on the use of Pioglitazone and Rosiglitazone indicated that some of these side effects are common (fluid retention, congestive heart failure, and bone fractures), thus suggesting that these effects were mediated by PPARγ [56,57]. Conversely, a meta-analysis, carried out in other clinical studies, demonstrated that Rosiglitazone had negligible effects on cardiovascular events like infarction or heart failure, whereas Pioglitazone reduced cardiovascular disease risk [44,58,59]. Moreover, a relevant risk of causing bladder cancer was evidenced in Pioglitazone-treated patients, while no carcinogenic effect was detected with Rosiglitazone [60,61]. Troglitazone, in contrast, was associated with hepatotoxicity, an effect not revealed with Rosiglitazone or Pioglitazone [62,63]. Altogether these findings strongly suggest that part of the effects of TZDs were attributable to the primary target of the drugs (PPARγ), the adverse effects were probably generated from the secondary targets of TZDs. For example, it has been reported that PPARγ full agonists, like TZDs, can interact with other nuclear receptors or functional proteins causing additional side effects [64,65,66]. It is conceivable that a differential distribution in the tissues and organs of the targets of PPARγ full agonists can lead to different effects, including adverse effects. It is also noteworthy to consider that many experiments to identify PPARγ full agonists have been carried out in in vitro systems (for example, cellular models or cell-free systems), but only a comparison with in vivo models could confirm or refute these results. The studies of PPARγ full agonist effects in animal models, including in mouse or, even more significantly, human models, should not neglect a deeper understanding of the distribution and concentration of PPARγ in the various tissues, as well as its own activation in the metabolism and in the regulation of vital processes.

**Figure 5:**
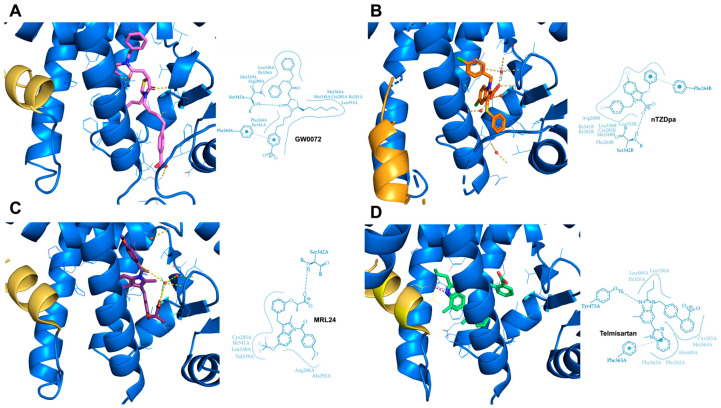
Crystal structures and PoseView maps highlighting key interactions of various ligands to the PPARγ LBD: (**A**) GWOO72 (PDB: 4PRG); (**B**) nTZDpa (PDB: 2Q5S); (**C**) MRL24 (PDB: 2Q5P); and (**D**) Telmisartan (PDB: 3VN2). H12 is shown in orange.

**Figure 6:**
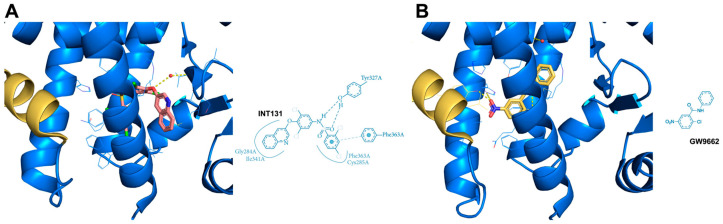
Crystal structures and PoseView maps highlighting key interactions of various ligands bound to the PPARγ LBD: (**A**) INT131 (PDB: 3FUR); and (**B**) GW9662 (PDB: 3B0R). H12 is shown in orange.

Bearing in mind the difficulty of fully managing these contradictory results, it was necessary to implement a strategy to distinguish between the beneficial and adverse effects caused by PPARγ full agonists. With this aim, many efforts have been conducted to investigate new PPARγ ligands, permitting the isolation and identification of the primary PPARγ target genes that cause beneficial consequences in controlling glucose resistance from those responsible for adverse side effects. Several molecules capable of partially activating PPARγ-mediated gene transcription were identified and named PPARγ partial agonists to distinguish them from full agonists [67]. Partial agonists do not usually occupy branch I of the PPARγ LBD pocket, which is surrounded by the H3, H5, H11, and H12 helices. Instead, they often enter branch II (delimited by H2, H3, H6, and H7 and a β sheet) and may also occupy part of branch III (enclosed by H2, H3, and H5 and a β sheet). The common feature of these different partial agonists consists in the exclusion of the fluctuating H12 helix, resulting in no contact with the H12 residues unlike full agonists. Most of the partial agonists prefer to occupy the space between the H3 helix and β-sheet, interacting with specific amino acid residues belonging to these two structures and, consequently, preferentially stabilizing the β-sheet region rather than the H12 helix [68]. Data obtained by nuclear magnetic resonance (NMR) spectroscopy and hydrogen–deuterium exchange (HDX) mass spectrometry indeed suggest that other PPARγ protein domains may play a role in the partial agonist-mediated receptor activation. Specific co-regulators, recruited by partial agonist binding, recognize the new interacting surfaces on PPARγ, inducing, in such a way, a partial or, more precisely, graded transcription, which is directed at a restricted set of PPARγ target genes. A seminal study carried out by Oberfield’s group in 1999 identified one of the first PPARγ partial agonisst, reported as GW0072 from the thiazolidine family (Figure 5A) [69]. The crystal structure of the PPARγ LBD/GW0072 complex clarified that this partial agonist occupied the region close to the H3 and H7 helices and the β-sheet, without interacting with residues H323, H449, and Y473 present on the inner surface of H12. This lack of interaction prevented the conformational changes in the H12 helix necessary for its stabilization. Similar binding patterns to the PPARγ LBD have been observed for other classes of partial agonists. nTZDpa (Figure 5B) and MRL24 (Figure 5C), belonging to the indole family compounds, interact with H3 through hydrogen bonds with Cys285 and Arg 288 and with the β-sheet region through hydrogen bonds with Ser242 and Ile341. These interactions allow them to occupy branches II and III of the PPARγ LBD pocket rather than branch I, stabilizing helix H3 and the β-sheet, resulting in a partial activation of the PPARγ pathway [70]. Telmisartan, an angiotensin II type I receptor inhibitor used to control blood pressure and treat cardiovascular diseases, is another example of a PPARγ partial agonist [71]. This molecule from the benzimidazole family binds to PPARγ in a manner similar to Rosiglitazone, wrapping around the H3 helix and making contact with the H3 helix through one of its benzimidazole group (Figure 5D). Unlike nTZDpa and MRL24, Telmisartan occupies branch I of the PPARγ LBD pocket rather than II and III, making a weak contact with H12. Although Telmisartan forms a hydrogen bond to the H12 residue Tyr473, this interaction is weaker than that brought about by Rosiglitazone due to the different distances between the groups. As a result, Telmisartan is unable to stabilize the dynamic H12 helix and to stimulate a full transcriptional activity, categorizing it as a partial agonist of PPARγ. INT131, a novel partial agonist of PPARγ from the sulfonamide family, fills the pocket space by inserting itself at the boundary of branches I, II, and III, partially wrapping around the H3 helix (Figure 6A) [71]. INT131 weakly binds to Tyr327 and makes hydrophobic contacts with Met364 of H3, Leu330 of H5, and Ile341 of the β-sheet. Altogether, these investigations demonstrated that full PPARγ agonists, such as TZDs, utilize an H12-dependent mechanism to recruit co-regulators and modulate the transcriptional response. In contrast, PPARγ partial agonists, such as GW0072, MRL24, Telmisartan, INT131, and others that have not been mentioned here, use an H12-independent mechanism to stimulate the transcription of target genes through the involvement of specific transcriptional co-modulators [72,73].

**Figure 7:**
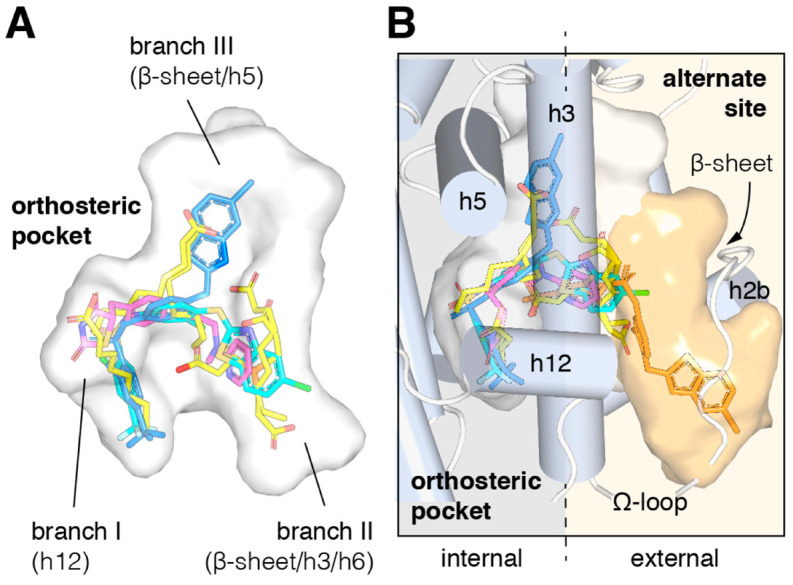
Structural locations of PPARγ orthosteric pocket and alternate site. (**A**) The T- or Y-shaped orthosteric pocket can accommodate one or more natural ligands such as nonanoic acid (C9; yellow sticks; PDB: 4EM9) and synthetic ligands such as Rosiglitazone (pink sticks; PDB: 4EMA) or T2384 (light and dark blue sticks representing different crystallized binding modes; PDB: 3K8S, chains A and B, respectively). (**B**) The orthosteric pocket (the white pocket surface) is completely enclosed within the alpha helical sandwich fold of the ligand binding domain (LBD). Ligands such as T2384 (orange sticks; PDB: 3K8S, chain B) can also bind to a solvent-accessible alternate site (the orange pocket surface) distinct from the orthosteric pocket, structurally defined as the region between helix 3 (H3) and the flexible Ω-loop (the dotted line separating the region internal to the LBD with a gray background and external to the LBD with a light orange background). From “Cooperative cobinding of synthetic and natural ligands to the nuclear receptor PPARγ” by Shang et al. (2018) eLife [81]. This article is distributed under the terms of the Creative Commons Attribution License, which permits unrestricted use and redistribution provided that the original author and source are credited “CC BY 4.0”.

The studies on natural or synthetic PPARγ ligands, both full and partial agonists, carried out through crystal structures analysis, strongly support the idea that these molecules differentially occupy the three branches of the PPARγ LBD pocket. In addition, it has been largely demonstrated that these ligands usually share common spaces in the PPARγ LBD pocket, being in competition for the occupancy of it [74,75,76,77,78,79]. Biological competition assays in the presence of GW9662, an irreversible PPARγ antagonist, have been instrumental in elucidating the behavior of PPARγ ligands and the related biological consequences. GW9662 covalently binds to Ser285 of the H3 helix, a key residue in branch I of the PPARγ LBD pocket, thereby causing the exclusion of any other ligand from occupying this site (Figure 6B). Numerous natural and synthetic PPARγ agonists have been, indeed, identified and characterized for their ability to occupy the same canonical site in branch I of the T- or Y-shaped PPARγ LBD pocket [75,76,77,78,79]. The use of GW9662 or other PPARγ antagonists like T0070907 in biological competition experiments has provided significant insights into the interactions between PPARγ and new ligand molecules. These studies have elucidated the functions of full or partial agonists in the activation of PPARγ-mediated gene transcription and their beneficial effects on the metabolism, cell proliferation, and other vital processes. Recent work by Hughes’s group demonstrated that PPARγ ligands, in addition, might interact not only with the canonical site but also with an alternate or allosteric site within the PPARγ LBD (Figure 7) [80,81]. This allosteric site partially overlaps with the canonical cavity near the β-sheet belonging to branch II but is more specifically located in the solvent-exposed space near the H3 helix, limited by the Ω-loop. Three interaction modes with the alternate site by the PPARγ ligands have been proposed. Firstly, a ligand that binds to the canonical (orthosteric) site may also bind to the alternate site, but at lower concentrations. Studies on the synthetic PPARγ ligands MRL20 and MRL24 revealed a differential modulation of these two sites in the LBD pocket, with a higher affinity (nM) for the canonical site versus a lower affinity (μM) for the alternate site. This differential binding may generate a more regulated biological response by these synthetic PPARγ ligands. In the second binding mode, the alternate or allosteric site becomes relevant when the canonical or orthosteric site is occupied by a PPARγ antagonist (GW9662 or T0070907) [76]. Indeed, daidzein, a PPARγ agonist, displayed a stronger neuroprotective effect through ligand binding independent activation when the canonical site in the PPARγ LBD pocket was blocked by a PPARγ antagonist [82]. The third model involves endogenous ligands, such as oxidized fatty acids, which covalently bind to the canonical site. In this scenario, the alternate site is also targeted by the same ligands, leading to allosteric control by these PPARγ ligands, thus increasing the beneficial effects in terms of anticancer properties [83]. This regulatory mechanism is often observed in cancer cells, which increase lipid biosynthesis to sustain their proliferation. Binding to the alternate site of PPARγ by fatty acids, produced by de novo biosynthesis or massively derived from diet, may support anti-cancer effects. The existence of two different sites within the PPARγ LBD pocket is plausible, given that the ligand binding domain of PPAR is larger than that of other nuclear receptors and can accommodate more than just one molecule. The presence of a second functional binding site in the large cavity of the PPAR receptor binding domain could reasonably explain the side effects, especially those from synthetic ligands as partial PPAR agonists competing with full agonists such as Rosiglitazone. Although it is intriguing that there is a regulatory site, i.e., an alternate site, that can influence the structure and function of PPARγ through the binding of new ligands, this hypothesis, however, still remains under investigation and not fully accepted. Few works have demonstrated the concomitant occupancy of both canonical and alternate sites by PPARγ ligands [74,84]. Further studies will be necessary to better explain how multiple molecules can bind to PPARγ, the mechanisms inducing conformational changes in the receptor structure, and the specific and differential biological effects these interactions provoke.

Recently, we have focused on determining the specific biological features of Cladosporols, a family of compounds acting as PPARγ ligands in regulating cell proliferation and migration in cancer. The study of these natural molecules, extracted from *Fungi*, furnished the clear demonstration that much can be learned from them and from other potential PPARγ ligands. This research promises to reveal new modes of binding and generate new hypotheses to explain the differential ability of promoting the activation or repression of the PPARγ-dependent gene expression program.

## 4. Natural PPARγ Ligands: Cladosporols as the Model of a PPARγ Ligand

**Figure 8:**
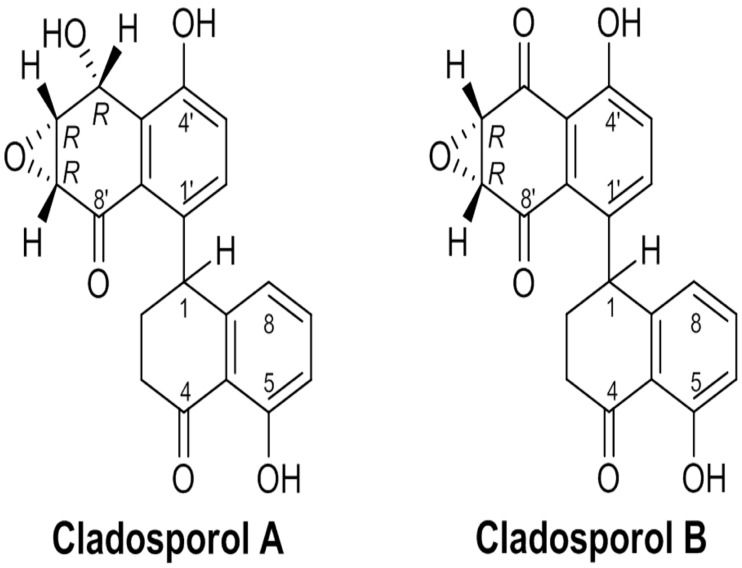
Chemical structures of Cladosporols A and B.

Cladosporols A and B, part of a small family of compounds, have been identified and isolated as secondary metabolites of *Cladosporium tenuissimum* (Figure 8) [85,86]. The selected fungal strain ITT21, derived from the destructive mycoparasite *Cladosporium tenuissimum* Cooke, was recovered from *Cronartium flaccidum* aeciospore samples collected in 1996 on *Pinus pinaster* in Tuscany (Italy) [85]. This fungus effectively controls the development of diseases in the field by parasitizing spores of rusts and other harmful plant fungal pathogens [86]. Cladosporols almost totally inhibit spore germination in various rust fungi, such as *Cronartium flaccidum*, *Melampsora pinitorqua*, and *Uromyces appendiculatus*, and decrease the radial growth of non-rust fungi, as in colonies of a phytopathogenetic fungi, like *Cercospora beticola*, *Botrytis cinerea*, and *Alternaria alternata*, and among *Oomycota*, *Phytophtora erytroseptica*, *Phytophtora cinnamomi*, and *Phytophtora capsici*. They also influence the growth of human pathogenetic strains of *Candida* sp. The antifungal properties of Cladosporols is likely to be associated with the intrinsic toxicity of the 2-tetralone chromophore and their highly reactive functionalities, such as the epoxy–alcohol moiety, which shares structural features with (+)-isoepoxidon, a well-known β-1,3-glucan biosynthesis inhibitor [87].

**Figure 9:**
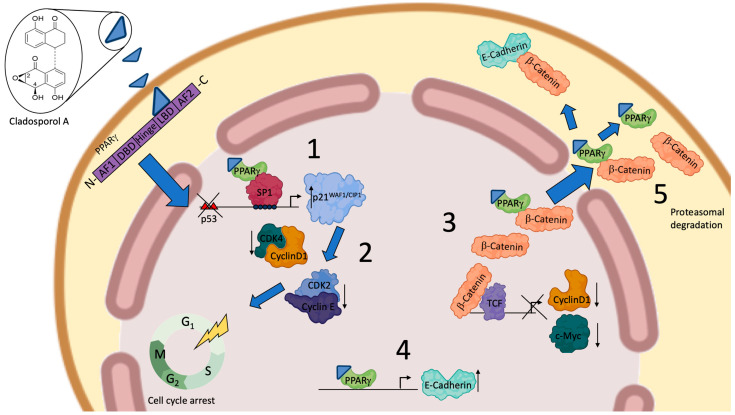
Cladosporol A actions regulating cell proliferation in colon cancer-derived cells. (1) Increase in p21waf1/cip1 transcription mediated by PPARγ and Sp1. (2) Down-regulation of cyclin D1, cyclin E, CDK2, and CDK4 expression and their phosphorylation activities. (3) Down-regulation of the β-catenin-dependent target gene transcription, i.e., cyclin D1 and c-myc. (4) PPARγ-dependent increase in E-cadherin mRNA transcription. (5) Proteasomal degradation of PPARγ and β-catenin.

**Figure 10:**
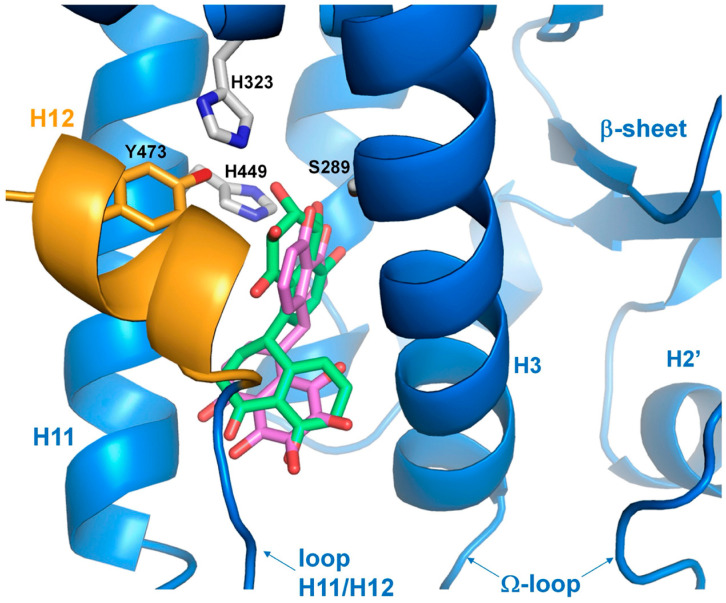
Docking of Cladosporol A (green stick) and Cladosporol B (violet stick) into the PPARγ LBD. The Ω–loop, a flexible loop region between H2 and H3, and β-sheet region of the LBD are displayed.

In our initial studies, we showed that Cladosporol A inhibited colorectal cancer (CRC) cell proliferation, particularly in HT-29 cells, by inducing a G1 phase arrest. This was accompanied by the simultaneous elevation in p21waf1/cip1 protein levels and the decrease in some cell cycle regulators (cyclin D1, CDK2, and CDK4) [88]. In addition, we identified Cladosporol A as a new PPARγ ligand and characterized its antiproliferative properties, demonstrating that it activated p21waf1/cip1 gene expression in an Sp1-dependent manner [89]. We also showed that Cladosporol A directed β-catenin to proteasomal degradation, reducing the nuclear protein level and, in turn, the transcription of oncogenetic target genes (c-myc and cyclin D1). β-catenin typically forms a complex with E-cadherin, generating cell–cell interactions and preserving the epithelial features of cells. We revealed that E-cadherin was a PPARγ target in HT-29 cells, further supporting the role of Cladosporol A as a new compound with antiproliferative and antimetastatic potential (Figure 9) [89]. Subsequently, we investigated the biological properties of Cladosporol B, the oxidized form of Cladosporol A. Cladosporol B also appeared to display antiproliferative and pro-apoptotic characteristics. Our studies demonstrated that Cladosporol B showed anticancer properties in HT-29 cells, which were not only dependent on the arrest of the cell cycle in the G0/G1 phase but also on a strong activation of programmed cell death [90]. The effects of Cladosporol B were more pronounced than those of Cladosporol A and appeared to be related to a different affinity for the PPARγ LBD, which is associated with a reduced potential for PPRE-mediated transactivation. The structural analysis of the binding of Cladosporols A and B to the PPARγ LBD domain revealed that Cladosporol B formed interactions with different amino acids compared to Cladosporol A and Rosiglitazone [90]. In particular, it emerged that, while Rosiglitazone was arranged perpendicular to the H3 helix, Cladosporol A assumed a north–south orientation parallel to the H3 helix, forming hydrogen bonds with the amino acid residues Y473, H449, and H327 with consequent stabilization of H12. On the contrary, Cladosporol B did not directly contact the H12 helix but stabilized the H3 helix through hydrophobic interactions with residues belonging to this helix (S289, F282, Q283, and Q286) (Figure 10) [90]. This different binding mode altered the LBD region structure, exposing new receptor surfaces and favoring their stabilization, which is necessary for the recruitment of the different transcriptional complexes. In other words, the different way of binding to the receptor with the recruitment of different co-regulators could lead to a distinctive activation of the genes eliciting specific biological functions [90]. Other compounds have been shown to display the same binding mode of Cladosporol B (when compared to the full agonist rosiglitazone) demonstrating reduced transactivation ability and stronger antiproliferative activity, typical features of partial agonists [91,92,93,94]. In detail, the weak interactions of these PPARγ partial agonists occur within the LBD pocket, with residues residing at a considerable distance from the H12 helix. The distance between Cladosporol B or similar partial agonists and the H12 helix is remarkable, thus preventing the binding to the three residues H323, H449, and Y473, which are characteristic of Rosiglitazone’s full agonism, and the maximal transcriptional activation of the target genes. Ligand docking assays suggested that the binding mode of these partial agonists rather implies the involvement of the side chains of the residues exposed from H3 and H11 and the 11/12 loop, preferably stabilizing H3 through hydrophobic bonds to the specific amino acids belonging to this helix (S289, F282, Q283 and Q286). The authors of these studies proposed designating this hydrophobic region in the branch I cavity of the PPARγ LBD pocket as the “diphenyl pocket”, suggesting that the stabilization of H3 is a specific feature and a crucial requirement of the partial agonists binding to the PPARγ LBD pocket. Beyond the classifications proposed by the different research groups, based on the type of binding and the specific branch of the pocket occupied by the different ligands (agonists, antagonists, or partial agonists), it is reasonable to conclude that most of the partial agonists do not occupy branch I of the pocket but prefer branches II and III between the H3 helix and the β-sheet, primarily stabilizing the H3 helix. Full agonists, instead, are closer to H12, making strong contacts with His 323, Tyr 473, and His 449. Some partial agonists may also occupy branch I of the pocket like rosiglitazone, but they no longer contact the His 323, Tyr 473, and His 449 triad. Although some contacts have been demonstrated for some partial agonists with H12, these interactions do not involve the triad, because the distance does not allow any bonding. In summary, we may conclude that partial agonists, like Cladosporol B, ureidofibrate-like derivatives, or chiral phenoxyacetic acid analogues, by moving away from the H12 helix and closer to the H3 helix, stabilize the latter instead of the former, thereby exposing novel interfaces with other hypothetical co-regulators of transcription different from those of full agonists [91,92,93,94].

## 5. Different Tissues, Different Biological Processes, but a Unique Target: PPARγ

**Figure 11:**
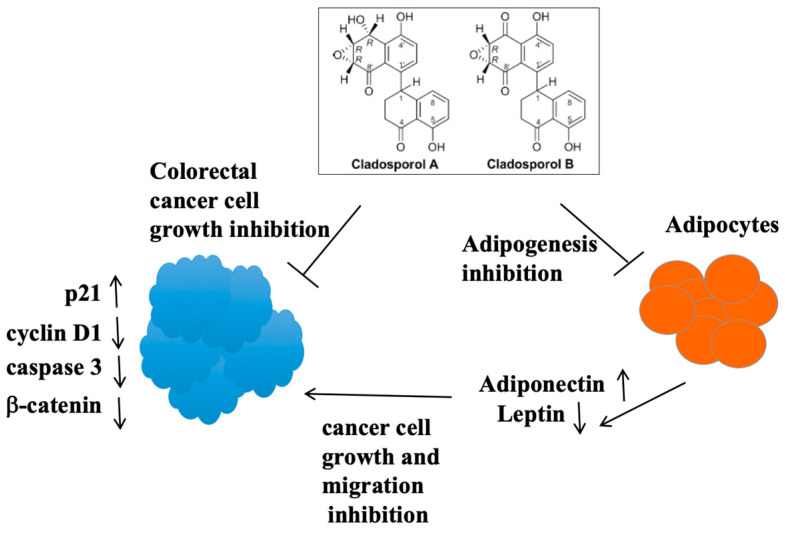
Cladosporol actions on 3T3-L1 cell differentiation and HT-29 CRC cell proliferation and migration.

Considering the aforementioned results, which elucidate the differential interaction of Cladosporols with PPARγ and the regulation of crucial biological processes, we further investigated the properties of Cladosporols A and B in 3T3-L1 cells through an in vitro murine model of preadipocytes committed to differentiation. Our study demonstrated that Cladosporols A and B inhibit adipogenesis in 3T3-L1 preadipocytes, which have the ability to differentiate into mature adipocytes. Specifically, both Cladosporols prevented the mitotic clonal expansion (MCE) of preadipocytes by arresting the cell cycle in the G0/G1 phase [95]. Using real-time polymerase chain reaction (PCR) and western blotting analyses, we also verified that the mRNA and protein levels of early and late adipogenetic markers (PPARγ and C/EBPα; aP2; and FASN) were significantly reduced by treatment with Cladosporol A or Cladosporol B (Figure 11).

Given the role of adipose tissue and its fatty acid depots in the tumor microenvironment, we investigated whether secreted factors (adipokines) from 3T3-L1 cells could influence HT-29 cell growth, invasion, and migration. We demonstrated that the proliferation and migration of HT-29 cells were inhibited by a medium derived from 3T3-L1 cells cultured in the presence of Cladosporol A or Cladosporol B. Specifically, we found an evident reduction in cyclin D1 and β-catenin and a simultaneous increase in p21waf1/cip1 expression in the HT-29 cells, indicating an evident cell cycle blockage in the HT-29 cells grown in the 3T3-L1 conditioned medium from cells exposed to either Cladosporols. Moreover, a significant stimulation of apoptosis was revealed and, in fact, a reduction in caspase-3 precursor levels was found in the HT-29 cells cultured in the conditioned medium from 3T3-L1 cells previously treated with either Cladosporols, suggesting an enhanced programmed cellular death. These findings prompted a molecular mechanism through which a dysregulated lipid metabolism in mature 3T3-L1 adipocytes and cancer promotion and progression in HT-29 cells could simultaneously be influenced by the use of the same therapeutic tool, i.e., Cladosporols [95]. Leptin and adiponectin are two adipokines produced by mature adipose tissue that integrate peripheral and central signals, but they also represent part of the milieu that stimulates the tumor microenvironment and thus regulates the signaling pathways related to cancer progression. We analyzed the presence of these important adipokines in 3T3-L1 cells and in the culture medium of undifferentiated and differentiated 3T3-L1 cells treated with Cladosporol A or Cladosporol B, respectively. Both Cladosporols reduced the leptin expression level in the cells and in the medium, while being responsible for a decreased adiponectin expression in the cells, but promoted adiponectin secretion in the medium [95]. These results strongly suggest that Cladosporols can regulate the production and availability of crucial hormones that differentiated adipocytes secrete in order to influence the biological properties of neighboring cells. Our findings suggested a Cladosporol treatment-dependent break in the crosstalk between 3T3-L1 mature adipocytes and HT-29 cells. The increased adiponectin synthesis and secretion in the medium from differentiated 3T3-L1 cells treated with Cladosporol A or Cladosporol B is responsible for the inhibition of HT-29 cell growth and migration (Figure 11) [95].

More recent data also show that PC-3 cells, derived from metastatic prostate cancer, were highly sensitive to the same regulation mediated by Cladosporol-treated 3T3-L1 adipocytes [96]. We firstly demonstrated that the inhibition of the growth of PC-3 cells, caused by both Cladosporols A and B, is mediated by PPARγ. This aligns with the results from other studies indicating that targeting the PPARγ2 isoform induces a strong apoptosis in the most aggressive prostate tumor cells [97,98]. The inhibition of PC-3 cell proliferation was, indeed, obtained through an increase in apoptosis as proved by the diminished expression of caspase-3, Bcl-2, and caspase-9, well-known apoptotic markers [96]. In addition, Cladosporols A and B, by regulating the expression of the proteins involved in cell proliferation and migration (β-catenin, E-cadherin, and MMP-9), were responsible for a reduced capability of the PC-3 cells in proliferating and migrating [96]. Cladosporols A and B modulated in the opposite manner the synthesis and secretion of leptin and adiponectin in the 3T3-L1 cells. Therefore, when PC-3 prostate cancer cells were cultured in the medium from Cladosporol-treated 3T3-L1 mature adipocytes, through an indirect mechanism both Cladosporols also influenced the proliferation and migration of the PC-3 cells [96]. Notably, the higher concentration of adiponectin protein levels in the medium culture of the Cladosporol-treated 3T3-L1 cells correlated well with the increase in the adiponectin receptor 1 (Adipo-R1) expression in the PC-3 cells cultured in this Cladosporol-conditioned medium from the 3T3-L1 cells [96]. These encouraging data interestingly indicated that adiponectin, poured off in the medium from the 3T3-L1 cells treated with Cladosporols, interrupts the dialogue between mature 3T3-L1 adipocytes and PC-3 cells in the tumor microenvironment, promoting a strong inhibition of cell growth and migration [96].

Lipid metabolism and cancer are strictly related, and, thereby, a dysregulated metabolism of lipids constitutes one of the most relevant hallmarks of cancer. To sustain the excessive proliferation rate, cancer cells continuously need fatty acids for membrane synthesis [99,100,101]. Prostate cancer cells, in particular, rely on increased de novo lipogenesis to meet the demands for membrane synthesis, redox balance, and the energy storage and activation of the intracellular pathways. We investigated whether Cladosporol A and Cladosporol B played a regulatory role in lipid metabolism. The exposure of PC-3 prostate cancer cells to Cladosporols dramatically inhibited lipid accumulation through a negative regulation of the enzymes directly involved in lipid metabolism, including acetyl-CoenzymeA carboxylase (ACC), fatty acid synthase (FAS), fatty acid binding protein 4 (FABP4), hydroxi-methyl-glutaril-CoenzymeA reductase (HMG-CoAr), and sterol responsive element binding protein 1 (SREBP-1) [96]. Our experiments showed that the Cladosporol treatments efficiently diminished the protein amount of these enzymes. Pre-exposure to GW9662 led to an additional reduction, indicating that the negative regulatory effect on lipid synthesis was due to Cladosporol binding to the PPARγ LBD pocket [96]. Interestingly, the reduction in the synthesis of the enzymes involved in SREBP-1-mediated cholesterol biosynthesis in PC-3 prostate cancer cells suggests that the inhibition of this pathway, due to the Cladosporol–PPARγ complex action, also impedes the de novo intratumoral biosynthesis of androgens. This is particularly relevant for prostate cancer re-growth, when patients are usually kept in a low androgen concentration condition, because they are under hormonal ablation therapy [102,103]. We may argue that the Cladosporol–PPARγ complex-mediated blockage of the metabolic pathway stimulating cholesterol synthesis also inhibited the intratumoral de novo androgen biosynthesis in PC-3 metastatic prostate cancer-derived cells.

Altogether, the results for cells deriving from different tissues allow us to state that, in distinctive cell contexts, Cladosporols can modulate different biological processes through the binding to the same molecular mediator, i.e., PPARγ [95,96]. However, the mode of binding to the PPARγ LBD pocket, which determines the choice of the transduction pathway that is primarily affected by the Cladosporol treatment, is quite different from that described for PPARγ full agonists. In accordance with this way of reasoning, the non-canonical binding of Cladosporols into the PPARγ LBD pocket could be the reason for their ability to inhibit adipogenesis in 3T3-L1 cells and also to interfere in the lipid metabolism dysregulation in metastatic PC-3 prostate cancer cells but, at the same time, to limit cell proliferation and migration in HT-29 CRC as well as in PC-3 cells through the inhibition of diverse pathways. At this point, a question is obligatory. Is Cladosporol A (or Cladosporol B) alone responsible for its actions directed towards multiple targets? The answer is likely no, because, after the interaction of the Cladosporols with the PPARγ receptor, a series of events follow, one after another, to realize the action of these natural PPARγ ligands. The stabilization of an interaction surface on the PPARγ structure, indeed, firstly needs a few, but more efficient, contacts between the functional groups of the ligands and amino acid determinants exposed in the PPARγ LBD pocket. It is reasonable to think that a perfect binding of Cladosporols, for example, to the PPARγ pocket is obtained through an adaptation that takes place across time. In a very interesting recent work, Shang and Kojetin proposed that the binding to the PPARγ pocket by ligands happens through a two-phase fit mechanism: an initial encounter complex is followed by a conformational change into the final bound state [104]. This mechanism could also be followed by Cladosporols in their searching for a more stable complex with PPARγ. Cladosporol A, indeed, enters branch I, sits parallel to the H3 helix, assuming a north–south orientation, and interacts with the amino acid residues Y473, H449, and H327 of the H12 helix with its consequent stabilization. On the contrary, Cladosporol B does not directly contact the H12 helix (which is located a little further away) but stabilizes the H3 helix through hydrophobic interactions with the residues belonging to this helix (S289, F282, Q283, and Q286) [91]. The diverse binding mode of the two Cladosporols, which arises as a result of searching for the best and most stable interaction, can be the cause of the LBD structure conformational change with the exposure of new receptor surfaces necessary for the recruitment of the new and specific transcriptional co-factors.

These co-factors are capable of directing the action of the Cladosporol/PPARγ complex towards the signal transduction pathways, which can discriminate and choose different molecular targets in different tissue types, thus generating multiple but specific effects. However, the identity of these co-regulators still remains unknown. In this scenario, covalent modifications of the PPARγ receptor, which may occur before or after the co-regulators’ recruitment, could also play a role in enabling new differential transduction pathways. Finally, the ability to direct the function of PPARγ towards new pathways could also be dependent on the concentration of the PPARγ isoforms in the target tissue and on the quality of the receptor itself. For instance, understanding which isoform is most prevalent in a specific tissue and which of these PPARγ isoforms preferably binds Cladosporols is an essential topic. Our experiments in 3T3-L1 mature adipocytes and HT-29 CRC and prostate PC-3 cells have clarified various aspects of Cladosporol’s role in modulating complex phenomena occurring in different tissues in the tumor microenvironment. At the same time, our studies have raised further questions that we must address to understand how these natural molecules achieve distinct actions in different tissues but with the common goal of inhibiting cell growth and migration in colon and prostate tumors.

## 6. Conclusions and Perspectives

We have not fully identified and finely elucidated the differential pathways through which Cladosporols regulate functions such as cell proliferation and migration and lipid metabolism in the cells derived from distinctive tissues. However, it is evident that this behavior of Cladosporols is partially due to their mode of binding to PPARγ. While our studies, alongside those of other investigators, proved a relationship between PPARγ and partial agonists in simple 2D cellular models and in cell-based assays, further experiments are needed to confirm these findings in in vivo or ex vivo models. The adoption of three-dimensional (3D) organotypic cell models could bridge the existing gap between the results coming out from the in vitro and in vivo results. Future research should focus on combining novel 3D phenotypic cell-based assays, using confocal or multiphoton microscopy with advanced analytical techniques such as mass spectrometry and a microarray for proteomic, transcriptomic, and metabolomics analyses. This integrative approach will greatly enhance our comprehension of the role of Cladosporols in tumor biology and aid in the development of new therapies targeting novel molecular players.

## Data Availability

Details regarding the results reported in the present review are available in the data bank (PubMed). These data refer to the papers on Cladosporols whose references are present at the end of the discussion (Refs. [88,89,90,95,96]).
